# Biotechnological Agents for Patients With Tumor Necrosis Factor Receptor Associated Periodic Syndrome—Therapeutic Outcome and Predictors of Response: Real-Life Data From the AIDA Network

**DOI:** 10.3389/fmed.2021.668173

**Published:** 2021-07-08

**Authors:** Antonio Vitale, Laura Obici, Marco Cattalini, Giuseppe Lopalco, Giampaolo Merlini, Nicola Ricco, Alessandra Soriano, Francesco La Torre, Elena Verrecchia, Antonella Insalaco, Lorenzo Dagna, Masen Abdel Jaber, Davide Montin, Giacomo Emmi, Luisa Ciarcia, Sara Barneschi, Paola Parronchi, Piero Ruscitti, Maria Cristina Maggio, Ombretta Viapiana, Jurgen Sota, Carla Gaggiano, Roberto Giacomelli, Ludovico Luca Sicignano, Raffaele Manna, Alessandra Renieri, Caterina Lo Rizzo, Bruno Frediani, Donato Rigante, Luca Cantarini

**Affiliations:** ^1^Department of Medical Sciences, Surgery and Neurosciences, Research Center of Systemic Autoinflammatory Diseases and Behçet's Disease and Rheumatology-Ophthalmology Collaborative Uveitis Center, University of Siena, Siena, Italy; ^2^Amyloidosis Research and Treatment Center, Fondazione Istituto di Ricovero e Cura a Carattere Scientifico (IRCCS) Policlinico San Matteo, Pavia, Italy; ^3^Pediatric Clinic, University of Brescia and Spedali Civili di Brescia, Brescia, Italy; ^4^Rheumatology Unit, Department of Emergency and Organ Transplantation, University of Bari, Bari, Italy; ^5^Department of Internal Medicine, Arcispedale Santa Maria Nuova-Istituto di Ricovero e Cura a Carattere Scientifico (IRCCS), Reggio Emilia, Italy; ^6^Clinical Pediatrics, University of Bari, Bari, Italy; ^7^Periodic Fever Research Center, Institute of Internal Medicine, Catholic University of the Sacred Heart, Fondazione Policlinico A. Gemelli, Rome, Italy; ^8^Division of Rheumatology, Department of Pediatric Medicine, Bambino Gesù Children's Hospital, Istituto di Ricovero e Cura a Carattere Scientifico (IRCCS), Rome, Italy; ^9^Unit of Immunology, Rheumatology, Allergy and Rare Diseases, Istituto di Ricovero e Cura a Carattere Scientifico (IRCCS) San Raffaele Hospital, Vita-Salute San Raffaele University, Milan, Italy; ^10^Rheumatology Unit, Santa Chiara Hospital, Trento, Italy; ^11^Division of Immunology and Rheumatology, Department of Paediatric Infectious Diseases, University of Turin, Regina Margherita Children's Hospital, Turin, Italy; ^12^Department of Experimental and Clinical Medicine, University of Florence, Florence, Italy; ^13^Division of Rheumatology, Department of Biotechnological and Applied Clinical Science, University of L'Aquila, L'Aquila, Italy; ^14^Department of Health Promotion Sciences Maternal and Infantile Care, Internal Medicine and Medical Specialties “G. D'Alessandro”, University of Palermo, Palermo, Italy; ^15^Rheumatology Section, Department of Medicine, University of Verona, Verona, Italy; ^16^Clinical Pediatrics, Department of Molecular Medicine and Development, University of Siena, Siena, Italy; ^17^Department of Medicine, Università Campus Bio-Medico di Roma, Rome, Italy; ^18^Medical Genetics, University of Siena, Siena, Italy; ^19^Genetica Medica, Azienda Ospedaliera Universitaria Senese, Siena, Italy; ^20^Rheumatology Unit, Department of Medical Sciences, University Hospital of Siena (Azienda Ospedaliera Universitaria Senese, AOUS), Siena, Italy; ^21^Department of Life Sciences and Public Health, Fondazione Policlinico A. Gemelli IRCCS, Rome, Italy; ^22^Università Cattolica Sacro Cuore, Rome, Italy

**Keywords:** tumor necrosis factor receptor-associated periodic syndrome, biologic therapy, personalized medicine, interleukin-1 inhibitors, tumor necrosis factor inhibitors, tocilizumab

## Abstract

**Objective:** To describe the role of biotechnological therapies in patients with tumor necrosis factor receptor associated periodic syndrome (TRAPS) and to identify any predictor of complete response.

**Methods:** Clinical, laboratory, and therapeutic data from 44 Caucasian TRAPS patients treated with biologic agents were retrospectively collected in 16 Italian tertiary Centers.

**Results:** A total of 55 biological courses with anakinra (*n* = 26), canakinumab (*n* = 16), anti-TNF-α agents (*n* = 10), and tocilizumab (*n* = 3) were analyzed. A complete response was observed in 41 (74.5%) cases, a partial response in 9 (16.4%) cases and a treatment failure in 5 (9.1%) cases. The frequency of TRAPS exacerbations was 458.2 flare/100 patients-year during the 12 months prior to the start of biologic treatment and 65.7 flare/100 patients-years during the first 12 months of therapy (*p* < 0.0001). The median duration of attacks was 5.00 (IQR = 10.50) days at the start of biologics and 1.00 (IQR = 0.00) days at the 12-month assessment (*p* < 0.0001). Likewise, a significant reduction was observed in the Autoinflammatory Disease Activity Index during the study period (*p* < 0.0001). A significant corticosteroid sparing effect was observed as early as the first 12 months of treatment both in the number of patients requiring corticosteroids (*p* = 0.025) and in the dosages employed (*p* < 0.0001). A significant reduction was identified in the erythrocyte sedimentation rate (*p* < 0.0001), C reactive protein (*p* < 0.0001), serum amyloid A (*p* < 0.0001), and in the 24-h proteinuria dosage during follow-up (*p* = 0.001). A relapsing-remitting disease course (OR = 0.027, C.I. 0.001–0.841, *p* = 0.040) and the frequency of relapses at the start of biologics (OR = 0.363, C.I. 0.301–0.953, *p* = 0.034) were significantly associated with a complete response. No serious adverse events were observed.

**Conclusions:** Treatment with biologic agents is highly effective in controlling clinical and laboratory TRAPS manifestations. Patients with a relapsing-remitting course and a lower frequency of flares at the start of treatment show more likely a complete response to biologic agents.

## Introduction

Tumor necrosis factor receptor associated periodic syndrome (TRAPS) is an autosomal dominant autoinflammatory disease caused by mutations of the *TNFRSF1A* gene, encoding for the tumor necrosis factor (TNF)-α receptor 1. This disease is clinically featured by recurrent inflammatory episodes mainly characterized by long-lasting fever associated with erythematous and typically migrans erythematous skin rash, myalgia sustained by monocytic fasciitis, abdominal and/or thoracic pain mainly owing to serositis, periorbital edema, and joint inflammatory involvement (1, 2). However, clinical spectrum and severity of TRAPS is widely varied and may depend on the different penetrance of gene mutations: high-penetrance variants generally manifest with early onset and severe disease; conversely, low-penetrance mutations are more frequently identified in adult-onset patients and often lead to less severe and atypical inflammatory manifestations with a low risk for amyloidosis (3, 4). Despite the protean clinical spectrum, based on data from the Eurofever Registry, Gattorno et al. have recently proposed clinical and genetic criteria aimed at classifying TRAPS patients depending on the genotype (confirmatory or not confirmatory) or, in cases with no data about genetic analysis, according to the presence or absence of specific clinical manifestations. However, these criteria were primarily built for research and scientific purposes rather than for a direct application in clinical practice (5).

Treatment of TRAPS patients is to be tailored according to disease severity and should be aimed at controlling inflammation during flares, avoiding recurrences of attacks and subclinical inflammation during intercritical periods and preventing long-term complications, including reactive AA amyloidosis. Ultimately, the therapy of TRAPS should allow an easy participation in daily activities and improvement of health-related quality of life (6).

The latest treatment recommendations for the management of TRAPS date back to 2015 (7) and suggest the use of the TNF-α inhibitor etanercept or interleukin(IL)-1 inhibitors in case of long-term disease activity possibly leading to AA amyloidosis or in the case of need for long-term corticosteroids use. In particular, etanercept was recommended for both inducing the improvement of clinical and laboratory parameters during flares (Level of Evidence, LoE = 2B; Strength of Recommendation, SoR = C) and to limit corticosteroid use in case of frequent attacks and/or subclinical inflammation between flares (LoE = 2B-3; SoR = C); however, loss of etanercept efficacy was reported as possibly occurring over time (LoE = 2B; SoR = C). Anti-IL-1 agents were indicated as beneficial in the majority of patients with TRAPS (LoE = 2B; SoR = B) also as maintenance treatment (LoE = 2B-3; SoR = C). Regarding other than biologic treatments, non-steroidal anti-inflammatory drugs (NSAIDs) were suggested as an attempt to provide symptom relief during flares (LoE = 3; SoR = D), while corticosteroids were indicated as useful in concluding disease attacks, although the beneficial effect of corticosteroids often decline, thus requiring increasing doses over time (LoE = 3; SoR = C). With respect to other than etanercept TNF-α inhibitors, adalimumab, and infliximab proved variable efficacy in case reports, including paradoxical flares after administration (8, 9). For these reasons, these agents were not recommended (LoE = 3; SoR = C).

More recently, clinical trials have corroborated the role of IL-1 inhibition as pivotal treatment approach for patients with TRAPS. In this regard, Gattorno et al. highlighted a rapid disease control and a sustained clinical benefit in patients with active TRAPS treated with the selective IL-1β antagonist canakinumab at the posology of 150 mg every 4 weeks, eventually to increase up to 300 mg every 4 weeks (10). These findings were later confirmed by De Benedetti et al. through a phase III clinical trial conducted on 46 TRAPS patients (11).

As loss of efficacy while on IL-1 inhibition has also been described (6, 12), posology adjustments and switching between different IL-1 blockers should also be considered; actually, these treatment strategies have proved to yield clinical benefit or recovery of efficacy in initially non-responsive or partially responsive patients (10, 11, 13).

In recent times, also the IL-6 antagonist tocilizumab has been reported to be a promising biologic agent for the treatment of TRAPS patients, but current experience is quite limited (14).

Based on these assumptions, the purpose of the present study is to describe the therapeutic use of biotechnological agents in patients with TRAPS managed in real-life contexts. In particular, biotechnological therapies have been assessed in the light of clinical and laboratory outcomes observed in different conditions of age at disease onset, penetrance of mutations and biologic line of treatment.

## Materials and Methods

### Patients and Data Collection

Patients suffering from TRAPS and treated with biologic agents during their clinical history (from 2000 to 2018) were retrospectively included in the study. Patients were enrolled in 16 Italian tertiary Centers participating to the AutoInflammatory Disease Alliance (AIDA) network; all patients had been diagnosed with TRAPS on the basis of genetic analysis (Sanger sequencing of *TNFRSF1A* gene or next generation sequencing) performed in subjects presenting with recurrent fever attacks and other inflammatory manifestations evocative of TRAPS. Before starting any biologic agent, all patients had undergone a careful laboratory and radiologic screening in order to exclude concomitant infections or neoplasms. Patients had been monitored at the start of biologics (baseline), 1 and 3 months later and every 3 months or in case of clinical need (safety concerns or severe relapses). Demographic, clinical, and therapeutic data were retrospectively collected by reviewing patients' medical charts.

Biologic agents used in TRAPS patients enrolled in the present study were: the IL-1 receptor antagonist anakinra; the fully humanized IgG1 monoclonal antibody specifically acting against IL-1β canakinumab; the fusion protein of the TNF-α receptor and the Fc region of human IgG1 etanercept; the chimeric anti-TNF-α monoclonal antibody infliximab; the fully humanized monoclonal antibody against human TNF-α adalimumab; and the humanized monoclonal antibody that binds specifically to both soluble and membrane-bound IL-6 receptors tocilizumab. The choice of the biologic agents employed had been dictated by the preference of the referring physicians on the basis of literature evidences at the start of the treatments (etanercept was the most widely used biologic agent before IL-1 inhibitors became the gold-standard therapy for TRAPS) and the specific needs related to the patient's clinical history (1, 7).

The primary aim of the present study was to describe the use of biologic treatments for TRAPS patients in real-life along with their therapeutic role in controlling clinical and laboratory manifestations. Secondary aims of the study were as follow: (i) to assess any impact on the frequency and the duration of inflammatory flares after the start of biologics; (ii) to evaluate the laboratory changes after the start of treatment including erythrocyte sedimentation rate (ESR), C reactive protein (CRP), serum amyloid A (SAA), and 24 h proteinuria; (iii) to evaluate the impact of biologics on disease activity and progression of long-term organ damage; (iv) to identify any decrease in the use of corticosteroids after the introduction of biologics; (v) to describe the safety profile; (vi) to assess the long-term survival of biologic treatment; (vii) to identify any predictors of complete response to biologic treatment; (viii) to identify any difference in the outcomes according to the different age at TRAPS onset (adult vs. pediatric disease onset), the penetrance of mutations (high- vs. low-penetrance), and any difference between patients treated with their first biologic and those treated with their second (or more) biologic agent.

The primary endpoints of the study consisted in the description of the therapeutic use of biologics in terms of agents employed, times at biologic introduction, dosages used, posology changes and switches made over time, reasons for withdrawal of biologics, response to treatment distinguishing among “complete response,” “partial response,” and “failure,” persistence of biologic treatment over time meant as drug retention rate (DRR).

The secondary endpoints consisted of the identification of a statistically significant decrease in: (i) the frequency of flares reported during the first 12 months from the start of biologic treatments compared to the preceding 12 months; (ii) the duration of inflammatory episodes observed at the 12-month follow-up visit compared to that observed at the start of biologic treatment; (iii) the number of patients treated with corticosteroids and the daily use of corticosteroids (mg/day, prednisone, or equivalent) among patients already administered steroids at 3-, 6-, and 12-month visits compared to the start of biologic treatment; (iv) ESR, CRP, SAA values, and the AutoInflammatory Disease Activity Index (AIDAI) score at the 1-, 3-, 6-, 12-month, and last follow-up visits compared to the start of biologic treatment. In this regard, the AIDAI score is a clinimetric parameter recently suggested for the evaluation of disease activity in patients with the four better known monogenic autoinflammatory diseases, including TRAPS (15). The TRAPS-related organ damage was assessed at the start of biologic treatment, at the 12-month visit and at the last assessment while on treatment with any specific biologic agent by using the Autoinflammatory Disease Damage Index (ADDI), a further clinimetric tool born to evaluate long-term damage and its changes in patients with monogenic autoinflammatory diseases (16).

Since the validation of ADDI dates back to 2018, this clinical instrument was calculated retrospectively (and not at the time of the visits) in many cases. The inclusion of musculoskeletal pain among items contributing to ADDI was allowed only when the duration of this symptom was longer than 6 months, as methodologically required by Ter Haar et al. (17). On the other hand, as the current version of AIDAI score cannot be calculated retrospectively, statistical analysis on this variable was restricted to 18 patients (22 treatment courses) starting treatments after 2014, year of publication of the AIDAI score (15).

The study protocol was conformed to the tenets of the Declaration of Helsinki and was approved by the local Ethics Committee of the University of Siena (**AIDA Project; Ref. N. 14951**). Informed consent was obtained from each patient for the retrospective evaluation of her/his medical charts.

### Definitions

*Complete response* was defined as complete and persistent control of both clinical and laboratory manifestations of TRAPS. Among patients with no complete response, *partial response* was meant as coexistence of the two following conditions: (i) decrease in clinical disease severity after the start of biologic treatment, corresponding to a reduction of at least 70% in the frequency of attacks, duration of flares, inflammatory markers and, when available, in the AIDAI score; (ii) a patient-reported improvement in clinical manifestations during flares for relapsing-remitting disease courses and outside of flares for chronic courses. *Failure* to biologic treatment was a definition reserved to patients not meeting criteria for complete response and partial response. The concept of *effectiveness* was defined as a complete or partial response with good safety profile (lack of serious adverse events). A *flare* was meant as an inflammatory episode characterized by increased temperature (>37.0°C) and at least another clinical TRAPS feature including typical or atypical rash, severe inflammatory myalgia, abdominal pain, thoracic pain, periorbital edema, serositis revealed at ultrasound, or other imaging instruments.

The frequency of attacks was estimated standardizing flares as number of events/100 patients-year, in order to overcome any bias related to the different length of follow-up.

Patients enrolled carried the following high-penetrance mutations: C43R (*n* = 2); C43Y (*n* = 1); C52Y (*n* = 2); C81Y (*n* = 1); C96R (*n* = 2); T50M (*n* = 7); L167_G175DEL (*n* = 1); S59P (*n* = 1); L167_G175DEL (*n* = 1). The low-penetrance mutations identified in the patients enrolled were as follow: c.143A>T (*n* = 1); D12E (*n* = 2); P46L (*n* = 1); R53G (*n* = 1); R92Q (*n* = 16); R104Q (*n* = 1); V95M (*n* = 1). Conversely, the penetrance of the mutations R394H (*n* = 1) and c.472+1G>A (*n* = 2) was not clear. Therefore, these mutations were not included in the analysis when searching for differences on the basis of penetrance of mutations.

*Relapsing-remitting disease course* was defined for patients suffering from acute attacks with fever, inflammatory involvement of any potential site (especially skin, muscles, abdomen, eye, joints, serous membranes, and lymph nodes) and increased inflammatory markers, but no clinical or laboratory signs of inflammation during intercritical periods. The *chronic disease course* was defined for patients with acute flares, but with symptomatic intervals between flares and/or inflammatory markers steadily elevated.

Pediatric onset TRAPS included those patients experiencing a disease onset when aged <16 years.

Pathologic proteinuria was defined as 24 h proteinuria >150 mg/24 h in adults and >5 mg/Kg/24 h in pediatric patients.

### Statistical Analysis

Descriptive statistics has included samples size, percentages, means, interquartile ranges (IQR), and standard deviations. Fisher exact test with 2 × 2 or 2 × 3 contingency tables was used for comparisons among groups in case of qualitative data. According to normality distribution assessed with Shapiro-Wilk test, comparisons for quantitative data among three or more groups were performed using Friedman test or Kruskall-Wallis test or ANOVA test, as required. Pairwise comparisons and *post-hoc* analysis in case of overall statistical significances were performed with unpaired two-tailed *t*-test or Mann-Whitney two tailed *U*-test or Wilcoxon test, as appropriate, and Bonferroni correction.

The DRR was assessed employing the Kaplan-Meier plot, with “time 0” corresponding to the start of biologic courses and the “event” being the discontinuation of therapy because of primary or secondary inefficacy, adverse events, or lack of compliance.

Predictors of complete response were identified by performing univariate analysis with “complete response” (yes/no) set as dependent variable; conversely, the following variables were set as independent: age at disease onset, age at the start of the biologic, penetrance of mutations, disease duration at the start of the biologic, disease course, number of flares during the 12 months preceding biologic introduction, use of NSAIDs at baseline, use of corticosteroids at baseline, control of disease manifestations using corticosteroids, previous use of colchicine, evidence of systemic amyloidosis, fulfillment of the Eurofever score for TRAPS classification (5), the line of biologic agent used, ESR, CRP, and SAA values observed during flares, presence of thoracic pain, skin rash, pericarditis, pleuritis, abdominal pain, myalgia, arthritis, lymphadenitis. Those variables showing an association to “complete response” with a significance level of 90% (*p* ≤ 0.1) at univariate analysis were used as independent variables for binary stepwise regression analysis, while “complete response” remained the dependent variable.

A significance level of 95% (*p* < 0.05) was considered for all statistical computations with the exception of univariate analysis, as specified above. Odds ratio (OR), the corresponding statistical significance and 95% confidence interval (C.I.) were provided for each predictor at binary stepwise regression analysis.

Statistical Package for Social Science (SPSS) 24.0 package was used for statistical analysis.

## Results

### Demographic and General Information

Forty-four TRAPS patients, corresponding to 55 biologic treatment courses, were included in the study. Table 1 summarizes demographic, clinical, and laboratory data from patients enrolled.

**Table 1:** Demographic features of patients enrolled; the table also summarizes TRAPS manifestations observed during flares along with laboratory markers recorded at the last flare before starting biologic treatments.

**Demographic and clinical information**	**Entire cohort (*n =* 44)**	**Anakinra (*n =* 26)**	**Canakinumab (*n =* 16)**	**Anti-TNF-α agents (*n =* 10)**
Age at disease onset, years, median (IQR)	9 (15.25)	15.5 (18.5)	5.5 (5.5)	7 (10.75)
Age at diagnosis, years, mean ± SD	31.5 ± 15.7	35.7 ± 15.3	27.9 ± 14.8	25.4 ± 11.8
Age at the start of biologic treatment, years, median (IQR) or mean ± SD	17 (28.5)	15 (29.5)	25.1 ± 16.6	13.7 ± 11.7
Age at enrollment, years, mean ± SD or median (IQR)	40.53 ± 17.3	45.32 ± 17.1	37.1 ± 18.2	40 (18)
Male/Female patients	23/21	13/13	9/7	6/4
Patients with pediatric onset-TRAPS, *n* (%)	31 (70.5)	16 (61.5)	14 (87.5)	8 (80)
Patients with adult onset-TRAPS, *n* (%)	13 (29.5)	10 (38.5)	2 (12.5)	2 (20)
High/low penetrance mutations	18/24	11/15	8/8	3/7
Family members with symptoms, *n* (%)	18 (40.9)	8 (22.2)	10 (62.5)	1 (10)
Relapsing-remitting disease course, *n* (%)	34 (77.3)	17 (65.4)	14 (87.5)	6 (60)
Chronic disease course, *n* (%)	10 (22.7)	9 (34.6)	2 (12.5)	4 (40)
Duration of flares, median (IQR) or mean ± SD	10 (8)	10.8 ± 5.4	9.6 ± 6.1	13.1 ± 7.5
Flares/Year, median (IQR)	4 (3)	5 (3)	3 (4)	6.1 ± 3.8
Amyloidosis at diagnosis (%)	5 (11.4)	4 (15.4)	2 (12.5)	1 (10)
**Clinical manifestations during flares**, ***n*** **(%)**				
Thoracic pain	13 (29.5)	9 (34.6)	2 (12.5)	4 (40)
Pericarditis	11 (25)	9 (34.6)	0 (0.0)	2 (20)
Pleuritis	3 (6.8)	1 (3.8)	1 (6.3)	0 (0)
Abdominal pain	29 (65.1)	13 (50)	13 (81.3)	7 (70)
Pharyngitis	13 (29.5)	10 (41.7)	0 (0.0)	2 (20)
Oral aphthosis	11 (25)	8 (30.8)	1 (6.3)	2 (20)
Skin rash	19 (43.2)	11 (42.3)	4 (25)	6 (60)
Limphoadenopathy	15 (34.1)	8 (30.8)	1 (6.3)	4 (40)
Myalgia	24 (54.5)	16 (61.5)	4 (25)	5 (50)
Arthralgia	29 (65.1)	20 (76.9)	5 (31.3)	9 (90)
Arthritis	13 (29.5)	7 (26.9)	3 (18.8)	3 (30)
Conjunctivitis	7 (15.9)	4 (15.4)	1 (6.3)	2 (20)
Periorbital pain	6 (13.6)	3 (11.5)	1 (6.3)	3 (30)
**Laboratory data**				
Eritrocyte sedimentation rate, mm/1 h mean ± SD	69.9 ± 32.3	42.1 ± 20.7	70.8 ± 78.0	49.8 ± 41.5
C-reactive protein, mg/dL (IQR)	5 (7.75)	2 (6)	3 (13.3)	12 (8)
Serum Amyloid A, mg/L (IQR)	66.5 (202)	128 (382)	154 (155)	100 (123)
Proteinuria, n (%)	6 (13.6)	5 (19.2)	1 (6.3)	1 (10)

*IQR, interquartile range; n, number of patients; SD, standard deviation; TNF, tumor necrosis factor*.

The following biologic agents had been employed: anakinra (*n* = 26), canakinumab (*n* = 16), tocilizumab (*n* = 3), TNF-α inhibitors (*n* = 10; 7 patients treated with etanercept, 2 treated with adalimumab, 1 treated with infliximab). Forty-four patients had undergone a single treatment course, while a second line biologic agent was used in 9 patients; one patient was also administered a third and a fourth biologic treatment course. All but 6 patients were under the age of 16 at the start of the biologic agents (anakinra in 4 cases; canakinumab in 2 cases).

Considering the whole number of biologic courses, the mean age at the start of biologic treatment was 34.45 ± 15.1 years; specifically, the median age at the start of the first biologic agent was 34.5 (IQR = 24.5) years, while the mean age at the start of the second (or more) biologic was 34.55 ± 10.61 years. The mean disease duration at the start of treatment was 21.48 ± 17.15 years when considering the whole number of treatment courses. More in detail, the disease duration was 20.65 ± 17.57 years for patients treated with their first biologic and 30 (IQR = 21.5) years for patients undergoing their second (or more) biologic agent. The median treatment duration in the whole number of treatment courses was 27.5 (IQR = 48.5) months, while the mean treatment duration was 37.22 ± 31.37 months among patients treated with their first biologic course and 46.64 ± 40.3 months among patients treated with their second (or more) biologic course.

### Response to Biologic Courses

Forty-one (74.5%) treatment courses led to complete response. In particular, complete response was observed in 17/26 (65%) courses with anakinra, 15/16 (94%) courses with canakinumab, 2/3 (66.7%) courses with tocilizumab, 5/7 (71.4%) courses with etanercept, 1/2 (50%) courses with adalimumab, and 1/1 (100%) course with infliximab.

Nine patients (16.4%) showed a partial response to biologic treatment: 4/26 (15%) treated with anakinra, 1/16 (6%) treated with canakinumab, 1 treated with tocilizumab, 2 treated with etanercept, and 1 treated with adalimumab.

Five (9%) patients experienced lack of efficacy, all of them treated with anakinra. These patients were subsequently treated with etanercept (*n* = 3, 2 experiencing complete response and 1 partial response) and adalimumab (*n* = 1, showing partial response), with the fifth patient lost at the follow-up.

No statistically significant differences were observed in the frequency of complete response based on different age at onset (10/14 among adult-onset cases and 31/41 among pediatric-onset patients, *p* = 0.51), different penetrance of mutations (19/29 among patients carrying low-penetrance mutations and 19/23 among subjects with high-penetrance variants, *p* = 0.67) and the line of biologic treatment (35/44 for first line cases and 6/11 for second or more biologic treatment courses, *p* = 0.12).

### Changes in Dosing

Regarding posology changes, an increase in the frequency of administrations was observed in 2 cases among the 41 treatment courses characterized by complete response (i.e., a patient treated with anakinra and a patient treated with canakinumab); conversely, a decrease in the dosage and/or in the frequency of administrations were possible in 13/41 (31.7%) cases experiencing complete response. In detail, the decrease of posology occurred in 6/26 patients treated with anakinra, 4/16 patients treated with canakinumab, 2/7 patients treated with etanercept, and 1/3 patients treated with tocilizumab.

Among patients showing a partial response, an increase in the frequency of administrations was observed in a patient treated with tocilizumab. Also, an increase in daily anakinra dosage was observed in 1 out of 5 patients experiencing treatment failure.

**Figure 1:**
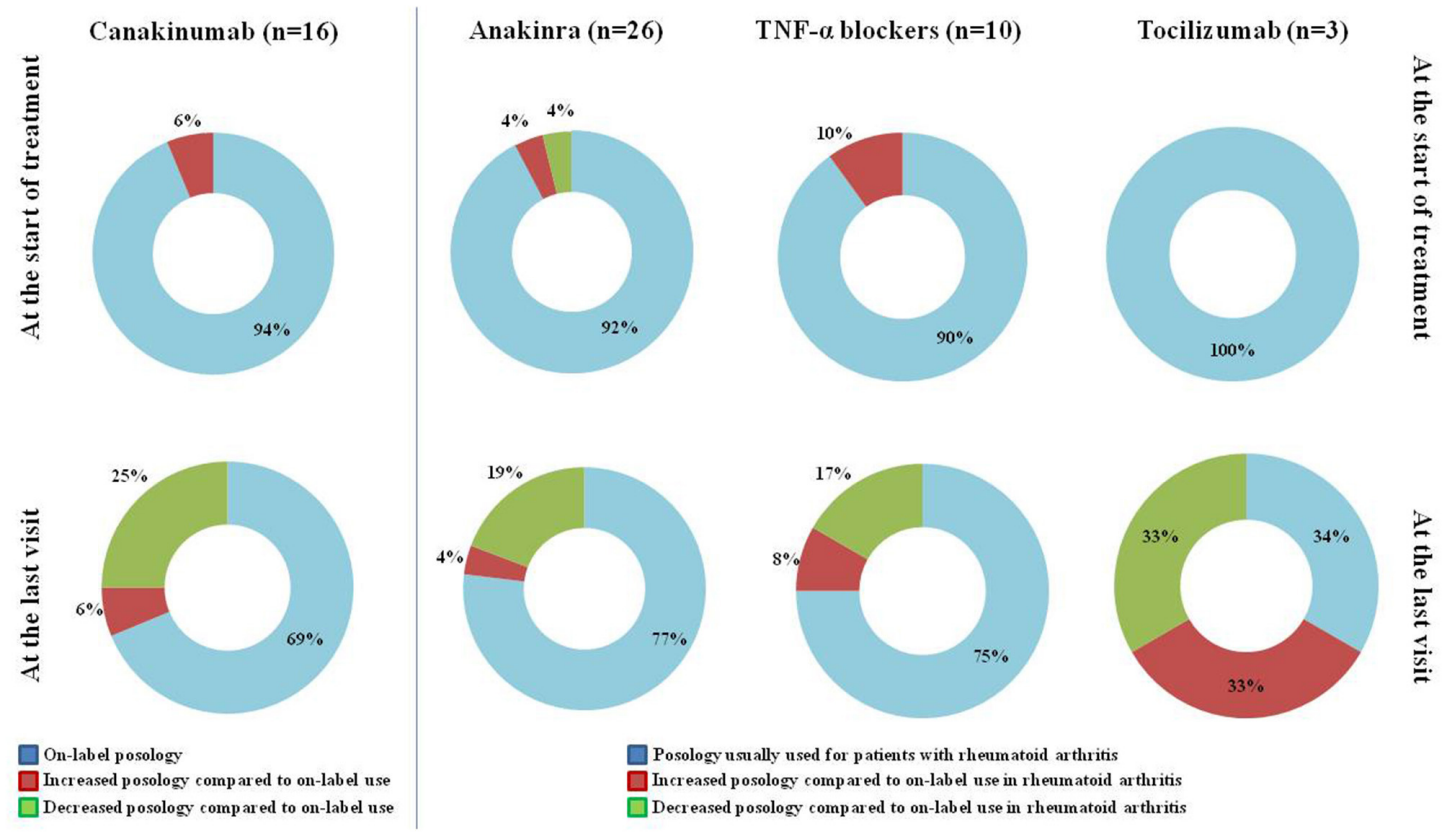
Posology strategies employed at the start of treatment and at the last assessment in the 55 treatment courses considered in the present study. The “*On-label*” posology for canakinumab corresponded to 150 mg/4 weeks (or 2 mg/Kg/4 weeks) to increase to 300 mg/4 weeks (or 4 mg/Kg/4 weeks) in case of unsatisfactory response to the former attempt. The “*Posology usually used for patients with rheumatoid arthritis*” along with the “*on-label use in rheumatoid arthritis*” were meant as 100 mg/day for anakinra, 50 mg/week for etanercept, 40 mg every other week for adalimumab, 3 mg/kg every 8 weeks for infliximab, 162 mg/week for tocilizumab. The 4 patients aged <16 years at the start of anakinra received the posology of 2–4 mg/Kg/day, which is considered “*on-label*” for patients with cryopyrinopathies, and were included in the group “*Posology usually used for patients with rheumatoid arthritis*.” TNF, tumor necrosis factor; *n*, number of patients (and of treatment courses).

Figure 1 provides details about the posologies employed for the different biologic courses observed in the present study, referring to the start of treatment and the last follow-up visit.

### Impact of Biologics on TRAPS Exacerbations

The frequency of TRAPS exacerbations was 458.2 flares/100 patients-year during the 12 months preceding the start of biologics and 65.7 flares/100 patients-year during the first 12-month of therapy (*p* < 0.0001). The median duration of attacks was 5.00 (IQR = 10.50) days at the start of biologics and 1.00 (IQR = 0.00) days at the 12-month assessment (*p* < 0.0001).

Among 44 biologic-naïve patients (25 treated with anakinra, 13 with canakinumab, 4 with etanercept, 1 with adalimumab, 1 with infliximab), the frequency of exacerbations corresponded to 468.2 flares/100 patients-year during the 12 months preceding the start of the biologics and 63.6 flares/100 patients-year during the first 12 months of treatment. A statistically significant reduction was observed in the number of flares after the start of biologic treatment (*p* < 0.0001). In this group, the median duration of attacks was 5.0 (IQR = 7.0) days at the start of treatment and 1.0 (IQR = 0.0) days after 12 months (*p* < 0.0001).

Among 9 patients (corresponding to 11 second, third and fourth biologic treatment courses) previously treated with other biologics, 418.2 flares/100 patients-year were recorded during the 12 months preceding the start of the treatment courses and 118.1 flares/100 patients-year during the first 12 months from the start of the biologics. A statistically significant reduction was observed in the number of flares after the start of biologic treatment (*p* = 0.018). In this group, the median duration of attacks was 4.0 (IQR = 33.69) days at the start of treatment and 1.0 (IQR = 2.25) days after 12 months (*p* = 0.005).

No statistically significant differences were observed in the decrease of both frequency (*p* = 0.28) and duration (*p* = 0.36) of flares between biologic-naïve patients and subjects undergoing their second (or more) biologic agent.

The frequency of inflammatory episodes among patients with adult-onset TRAPS was 485.7 flares/100 patients-year during the 12 months preceding the start of biologics and 14.3 flares/100 patients-year during the first 12 months of treatment (*p* = 0.003). The frequency of attacks in patients with early-onset TRAPS was 418.2/100 patients-year during the 12 months preceding the start of biologics and 80 flares/100 patients-year (*p* < 0.0001) during the 12 months thereafter. Among adult-onset TRAPS patients, the median duration of attacks was 5.00 (IQR = 14.75) days at the start of treatment and 1.00 (IQR = 0.00) days after 12 months (*p* = 0.002); among early-onset TRAPS patients, the median duration of attacks was 5.00 (IQR = 8.31) days at the start of treatment and 1.00 (IQR = 1.00) days after 12 months (*p* = 0.001). No statistically significant differences were observed in the decrease of frequency (*p* = 0.32) and duration (*p* = 0.13) of flares between adult- and pediatric-onset TRAPS.

Among subjects carrying a low-penetrance mutation, the frequency of inflammatory episodes was 603/100 patients-year during the 12 months preceding the start of biologics and 96.3/100 patients-year during the following 12 months (*p* < 0.0001). Among subjects with high-penetrance mutations, the frequency of flares was 386.95/100 patients-year during the 12 months preceding the start of biologics and 95.7/100 patients-year (*p* < 0.0001) during the subsequent 12 months. Among subjects carrying low-penetrance mutations, the median duration of flares was 5.00 (IQR = 10.00) days at start of treatment and 1.0 (IQR = 1.0) days 12 months thereafter (*p* < 0.001). Among subjects with high-penetrance mutations, the median duration of flares was 4.5 (IQR = 13.62) days at the start of biologics and 1.0 (IQR = 0.0) after 12 months (*p* = 0.003). No statistically significant differences were observed in the decrease of frequency (*p* = 0.50) and duration (*p* = 0.71) of flares between patients carrying high- and low-penetrance mutations.

### Clinimetric Features

**Figure 2:**
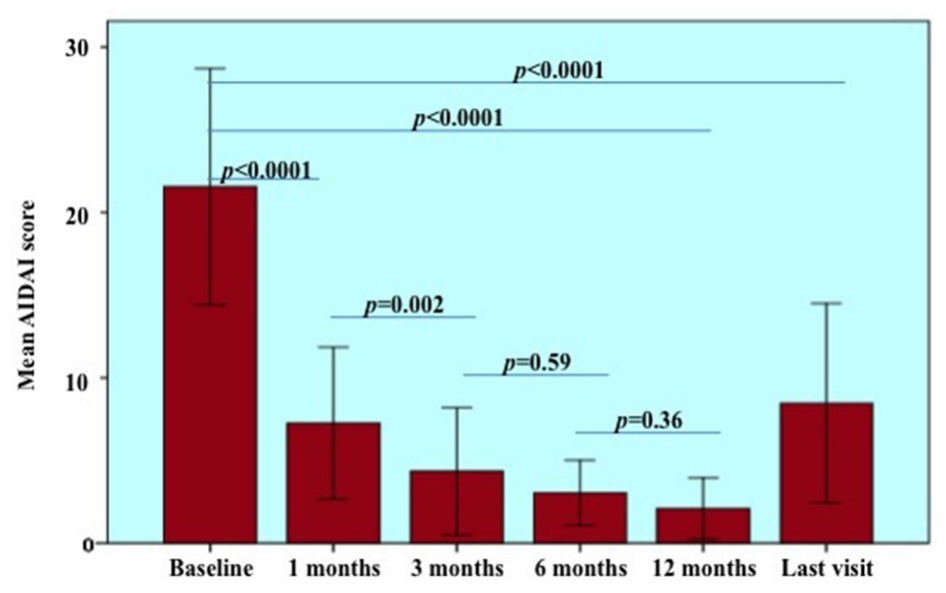
Autoinflammatory disease activity index (AIDAI) recorded at the start of treatment courses (baseline) and during the following visits. Statistical differences resulting from pairwise comparisons between different follow-up visits were provided to better detail the overall significance (*p* < 0.0001). Of note, statistical analysis on AIDAI changes was restricted to 18 patients (22 treatment courses) starting treatments after 2014, year of the AIDAI publication. Error bars refer to one standard deviation.

Considering treatment courses started after 2014, when the current AIDAI version was published (14), a statistically significant reduction was observed in the AIDAI score recorded at the follow-up assessments performed during the first 12 months of treatment (*p* < 0.0001). Figure 2 provides statistical details about changes in the AIDAI score observed at different follow-up visits. No statistically significant differences were observed in the decrease of the AIDAI score at 1-, 3-, 6-, and 12-month assessments according to the penetrance of mutations (*p* = 0.36, *p* = 0.42, *p* = 0.43, *p* = 0.85, respectively) and the different lines of biologic treatment (*p* = 0.70, *p* = 0.33, *p* = 0.31, and *p* = 0.85, respectively). The decrease of AIDAI score was significantly higher in adult-onset TRAPS (median decrease of 12.00, IQR = 24.00) than in patients with pediatric onset disease (median decrease of 5.00, IQR = 10.5) at the 1-month assessment, while no statistically significant differences were found at 3-, 6-, and 12-month assessments according to age at onset (*p* = 0.29, *p* = 0.54, *p* = 0.61, respectively).

None but two patients showed an increase in the ADDI score during the entire treatment period: in particular, one patient carrying the C43T mutation had been treated with canakinumab at the dosage of 150 mg every 4 weeks for 59 months; the second patient carried the D12E mutation and was treated with etanercept at the dosage of 50 mg/weekly for 14 months. On the contrary, chronic musculoskeletal pain contributing to the baseline ADDI score resolved during 15 (27.3%) treatment courses. In particular, if considering the resolution of this item, the ADDI score would decrease by one point in 7/26 (27%) anakinra treatment courses, 4/16 (25%) canakinumab treatment courses, 2/7 (29%) etanercept treatment courses, 1/3 (33.3%) tocilizumab course, and 1/2 (50%) adalimumab course. Seven out of 15 patients experiencing ADDI improvement carried high-penetrance mutations.

### Laboratory Parameters

**Figure 3:**
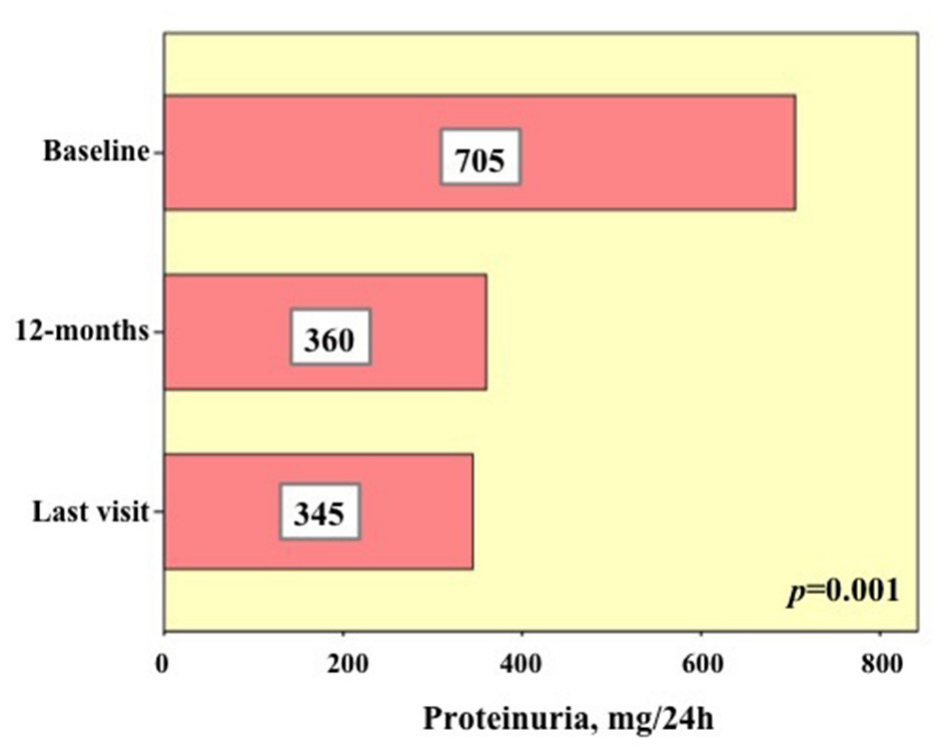
Details about 24 h proteinuria in six patients (eight biologic courses) presenting with pathologic proteinuria (i.e., >150 mg/24 h) at the start of biologic treatments (baseline), after 12 months and at the last assessment. The numbers added in the histograms indicate the median of the 24 h proteinuria values found in the eight biologic courses at the three time-points.

Six patients (eight treatment courses) presented proteinuria at the start of treatment. Two of these patients (3 treatment courses) presented end stage renal disease as soon as the start of the treatment. Anakinra had been used in 5 cases; canakinumab, tocilizumab and infliximab had been used each in one case. The median duration of the eight treatment courses was 22.5 (IQR = 34.25) months. None of the patients developed new end stage renal disease at the last follow-up assessment. Figure 3 shows the 24 h proteinuria at baseline, 12-month visit and at the last laboratory assessment while on the treatment courses. A statistically significant decrease was observed in the 24 h proteinuria during follow-up (*p* = 0.001).

**Figure 4:**
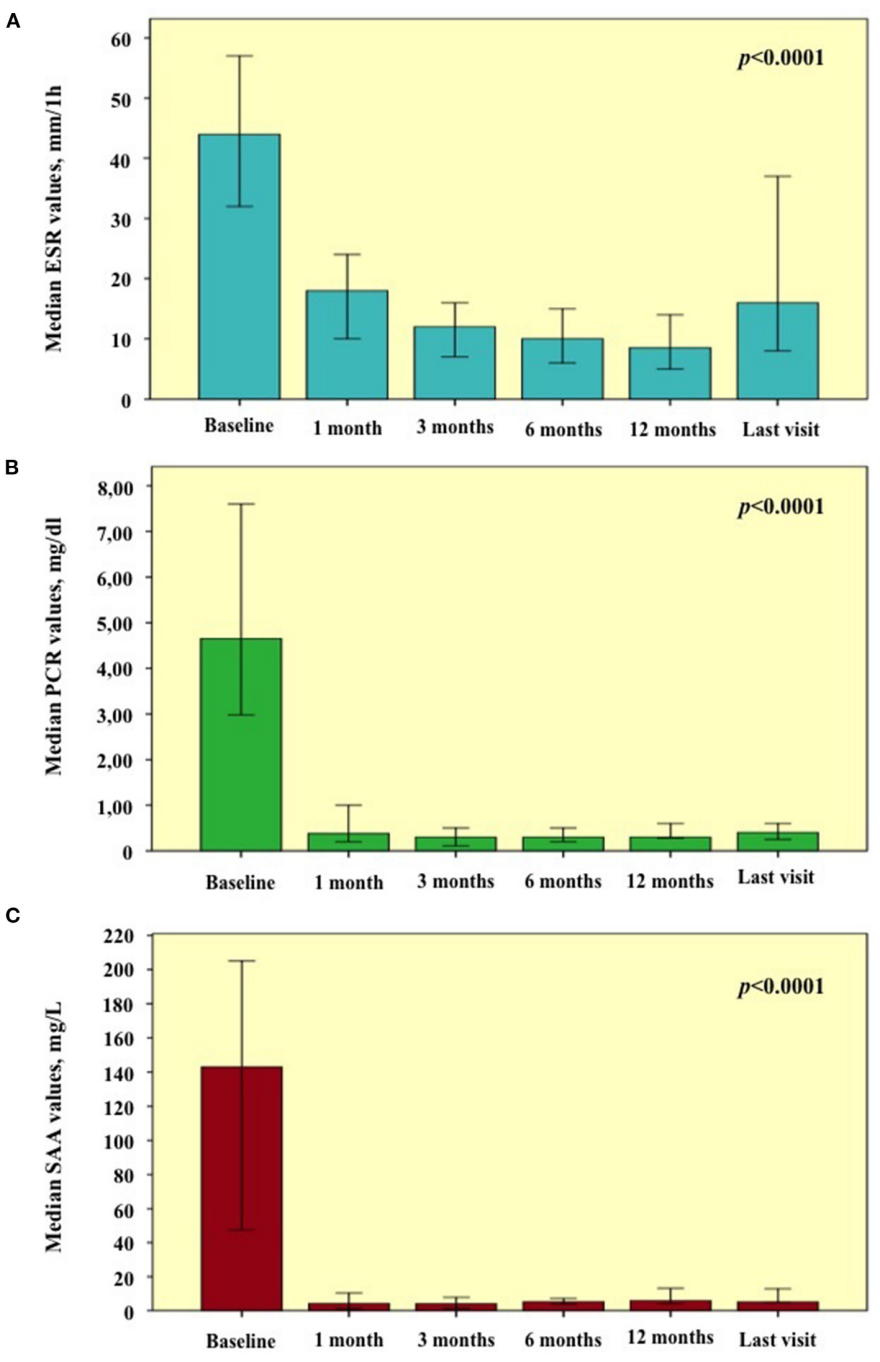
Laboratory inflammatory markers observed at the start of treatment courses (baseline) and during the following visits. Graphics are referred to **(A)** erythrocyte sedimentation rate (ESR); **(B)** C-reactive protein (CRP); **(C)** serum amyloid A (SAA). Error bars refer to one standard error.

Regarding inflammatory markers, a statistically significant reduction was found during the study period in the ESR (*p* < 0.0001), CRP (*p* < 0.0001), and SAA (*p* < 0.0001) values, as graphically reported in Figure 4. Table 2 provides ESR, CRP and SAA values referring to the different follow-up visits.

**Table 2:** Laboratory values of erythrocyte sedimentation rate (ESR), C reactive protein (CRP), and serum amyloid A (SAA) at the start of treatment courses and at the following visits.

	**ESR, mm/1h**	**CRP, mg/dl**	**SAA, mg/L**
Baseline	44 (53.25)	4.65 (11.1)	143 (334.4)
1-month assessment	18 (19)	0.38 (1.2)	4.18 (14.55)
3-month assessment	12 (16)	0.3 (0.7)	4 (10)
6-month assessment	10 (12)	0.3 (0.6)	5.2 (11.3)
12-month assessment	8.50 (12)	0.3 (0.6)	5.58 (13.4)
Last assessment	16 (35)	0.4 (1.8)	5.0 (28.1)

*Data are provided as median values and interquartile range in parentheses*.

### Impact of Biologics on Corticosteroids Intake

The number of patients taking corticosteroids significantly decreased during the first 12 months of treatments (*p* = 0.025); in detail, the number of patients administered corticosteroid were 26 (47.3%) at baseline, 17 (30.1%) at 3-month visit, 12 (21.8%) at 6-month visit and 8 (14.5%) at 12 month visit. The frequency of patients requiring corticosteroids decreased in a non-statistically significant manner at 3 month visit (*p* = 0.12); conversely, the number of patients treated with corticosteroids significantly decreased at 6-month (*p* = 0.009) and 12 month (*p* < 0.0001) assessments compared to baseline.

**Figure 5:**
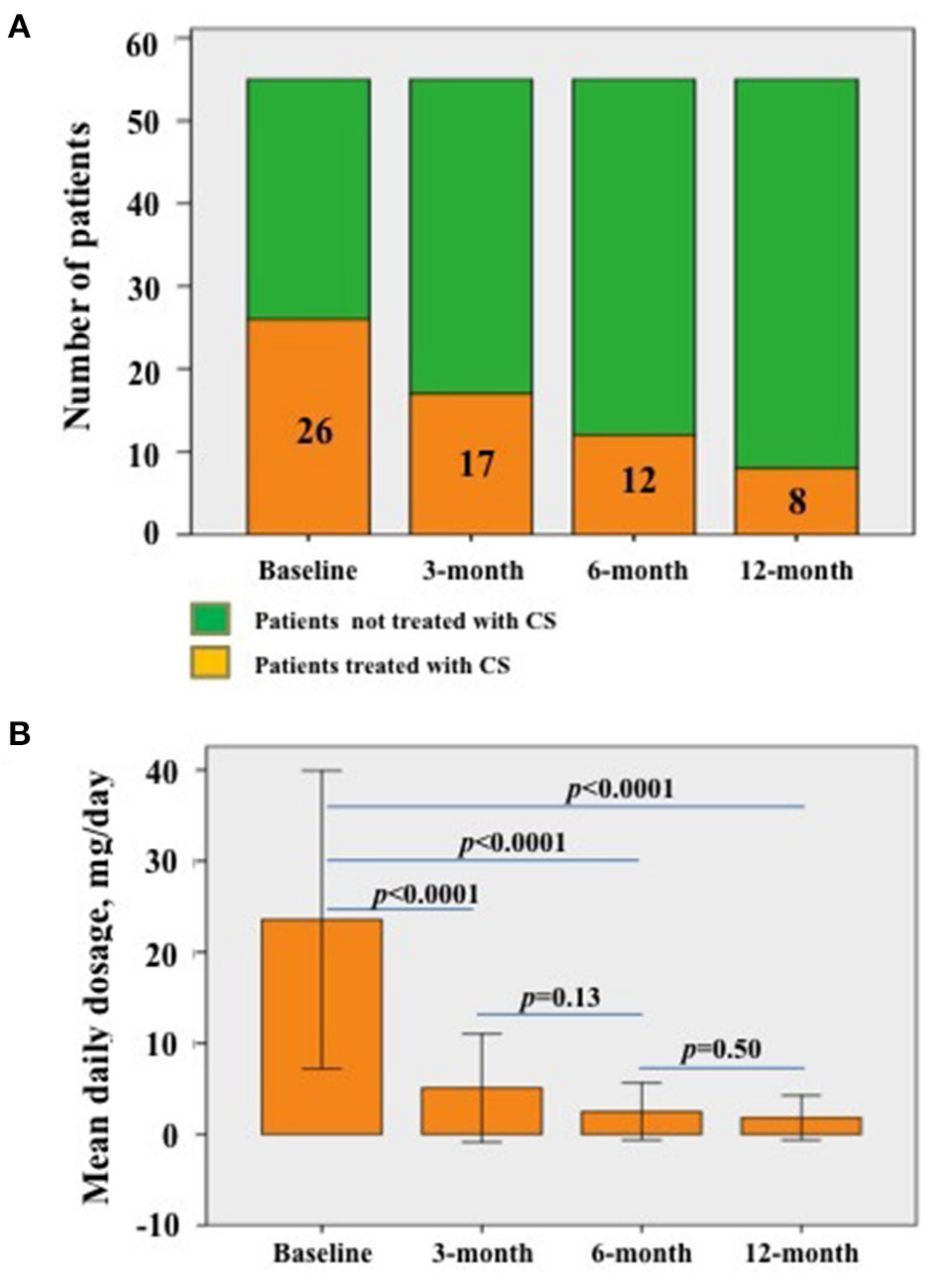
Details about corticosteroid administration. **(A)** Patients taking corticosteroids at the start of biologic treatments (baseline) and at the following visits; the decrease is statistically significant (*p* = 0.025). The numbers contained in the histograms refer to the specific number of patients taking corticosteroids at the corresponding visit. **(B)** Median daily corticosteroid dosage (as prednisone or equivalent) among patients already treated with corticosteroids at the different follow-up visits; the decrease is statistically significant (*p* < 0.0001). Statistical differences regarding the daily corticosteroid dosage between different follow-up visits have also been provided in the figure.; error bars in **(B)** refer to one standard deviation.

The median daily corticosteroid dosage employed was 20.00 (IQR = 17.5) mg/day at the start of treatment, 2.5 (IQR = 5.0) mg/day at both 3- and 6-month assessments, and 0.00 (IQR = 5.00) mg/day at the last follow-up visit. A statistically significant decrease was observed during the study period (*p* < 0.0001). Figure 5 shows details about frequency and amount of daily corticosteroid employment.

**Figure 6:**
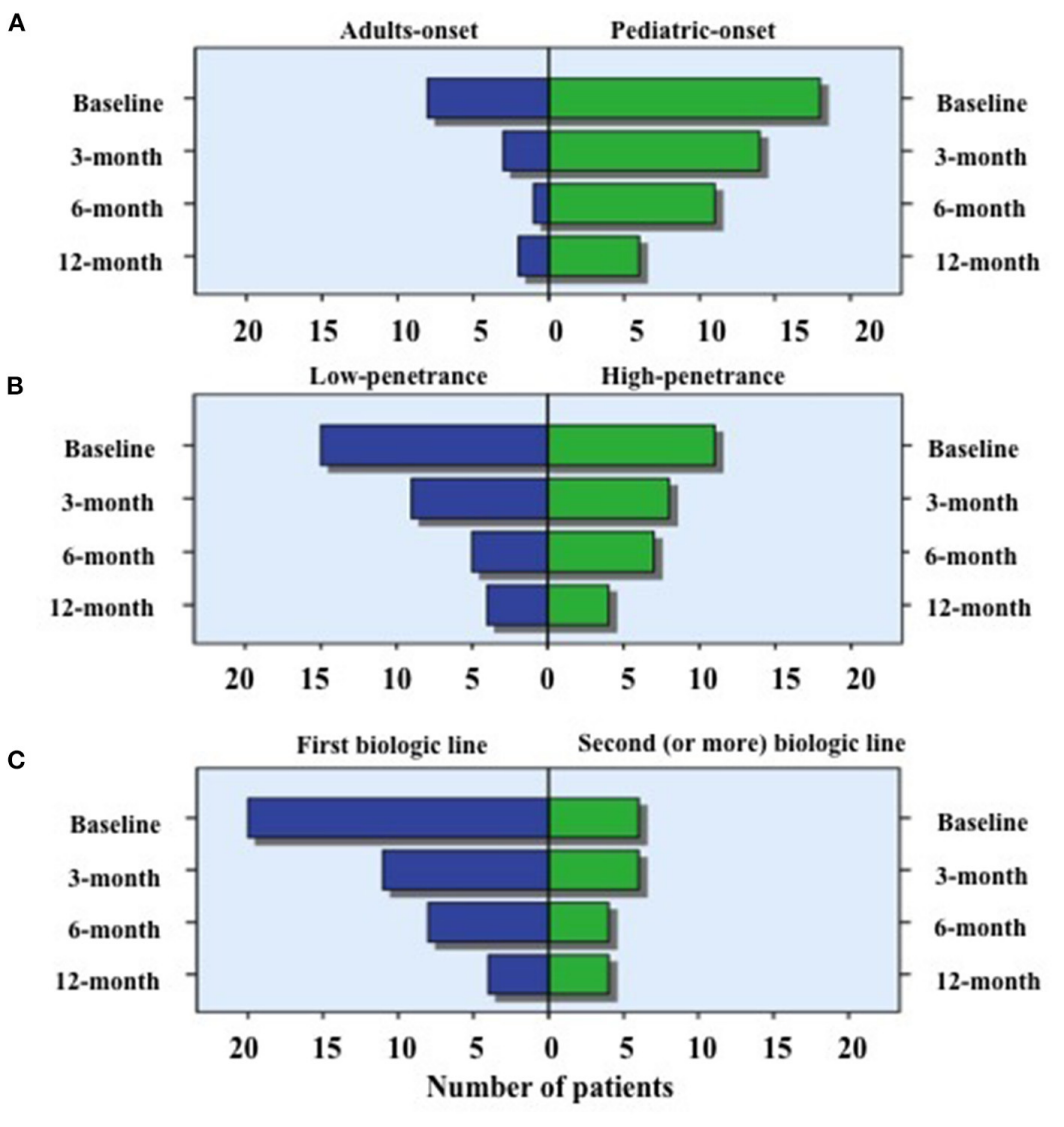
Use of corticosteroids in specific subgroups of patients. Number of patients requiring the use of corticosteroids at the different time-points, distinguishing biologic treatment courses according to: **(A)** the age at TRAPS onset (adult vs. pediatric onset); **(B)** penetrance of mutations (high- vs. low-penetrance) and **(C)** the line of biologic agents (first line vs. second or more line). As also reported in the text, the number of patients discontinuing corticosteroids was statistically significant higher in adult-onset TRAPS patients when compared to pediatric-onset patients at 3-month (*p* = 0.01) and 6-month (*p* = 0.02) assessments. Conversely, neither statistically significant difference was observed according to the different age at onset at the 12-month visit nor between subjects carrying low-penetrance and high-penetrance mutations or based on the biologic treatment line at the 3, 6, and 12-month assessments.

As graphically described in Figure 6, the number of patients discontinuing corticosteroids was statistically significant higher in adult-onset TRAPS patients at 3-month (*p* = 0.01) and 6-month (*p* = 0.02) assessments, while no statistically significant differences were observed at 12-month visit (*p* = 0.25) according to age at onset. No statistically significant differences were observed between subjects carrying low-penetrance and high-penetrance mutations in the frequency of patients suspending corticosteroids at 3-month (*p* = 0.32), 6-month (*p* = 0.23) and 12-month (*p* = 0.42) assessments compared to baseline. Similarly, based on the biologic treatment line, no statistically significant differences were observed in the frequency of patients suspending corticosteroids at 3-month (*p* = 0.35), 6-month (*p* = 0.29), and 12-month (*p* = 0.16) visits.

### Cause of Biologic Withdrawal and Safety Concerns

**Figure 7:**
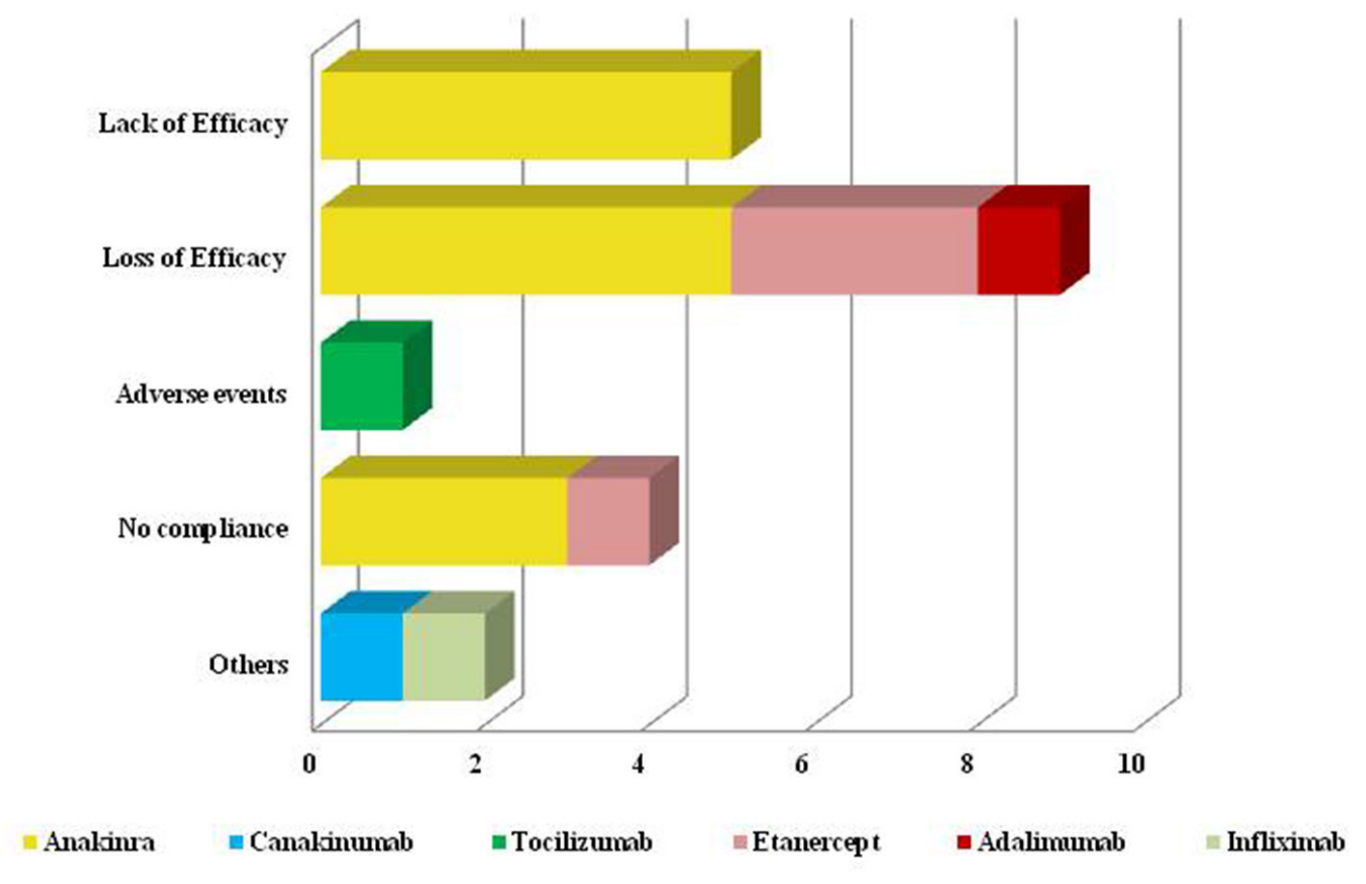
Reasons for biologic discontinuation. Graphical representation of reasons leading to biologic treatment discontinuation. As better explained in the text (*Cause of biologic withdrawal and safety concerns*), the term “Others” includes a patient experiencing the onset of inflammatory bowel disease during canakinumab treatment and a patient experiencing paradoxical flares after infliximab administrations.

Twenty-one patients had interrupted their treatment (12 patients administered anakinra, 1 canakinumab, 1 with tocilizumab, 5 TNF-α inhibitors) due to the reasons shown in Figure 7. The item “Others” included one patient experiencing the onset of a concomitant inflammatory bowel disease after 64 months of canakinumab treatment and one patient suffering from paradoxical flares after infliximab administrations. This last adverse event occurred after 18 months of complete response.

During the whole follow-up, 11 patients (20%) experienced adverse events. In additions to the already mentioned inflammatory bowel disease and paradoxical flare, 3 patients treated with anakinra suffered from injection site reactions; 1 patient developed pneumonia after 45 months of treatment with tocilizumab and 1 patient presented recurrent lower urinary tract infections while on treatment with canakinumab; mild thrombocytopenia and unilateral galactorrhoea were separately observed in 2 different patients administered tocilizumab. Amnesia and chest tightness were separately reported in 2 patients undergoing anakinra treatment.

### Persistence on Treatment

**Figure 8:**
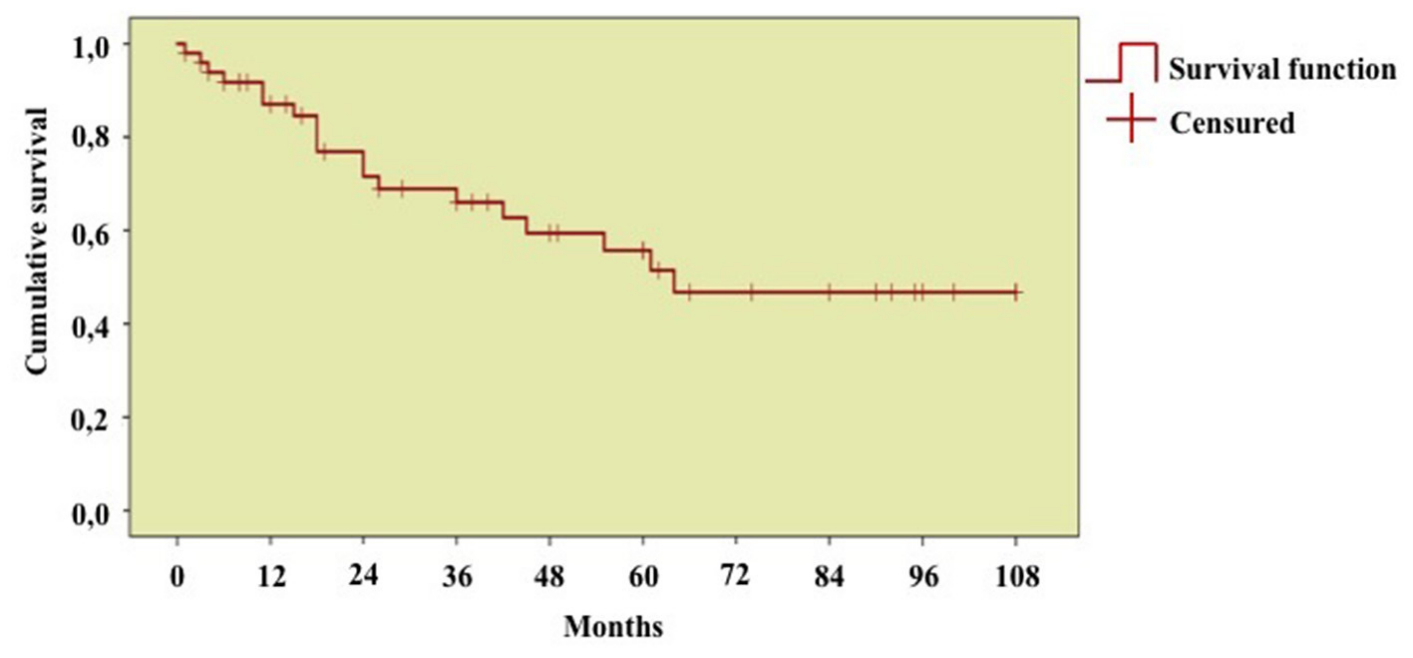
Long-term drug retention rate of biologic treatment courses. Kaplan-Meier survival curve describing the overall drug retention rate assessed on 55 treatment courses. Time 0” corresponds to the start of biologic courses and the “event” represents the discontinuation of therapy because of primary or secondary inefficacy, adverse events, or lack of compliance.

As shown in Figure 8, the DRR of biologic treatments assessed on the whole number of treatment courses was 87.0, 76.9, 66.0, 56.9, 55.7, and 46.8 at 12-, 24-, 36-, 48-, 60-, and 96-month assessments, respectively.

No differences were observed in the DRR of biologic agents according to penetrance of mutations (*p* = 0.11), age at disease onset (*p* = 0.29), and biologic line (*p* = 0.78).

### Predictive Factors of Complete Response

At univariate analysis, relapsing-remitting rather than chronic disease course (*p* < 0.0001) and disease duration at the start of the biologic (*p* = 0.01) were found to be predictors of complete response to biologic treatment. Likewise, the frequency of flares during the 12 months preceding biologic introduction (*p* = 0.06) and the control of disease manifestations with corticosteroids (*p* = 0.10) were associated to complete response with a *p* ≤ 0.1 and also entered multivariate stepwise regression analysis. Conversely, the following variables were not found to affect the complete response: age at onset (*p* = 0.76), age at the start of the biologic (*p* = 0.72), penetrance of mutations (*p* = 0.25), use of NSAIDs at baseline (*p* = 0.85), use of corticosteroids at baseline (*p* = 0.78), previous use of colchicine (*p* = 0.66), systemic amyloidosis (*p* = 0.23), fulfillment of the Eurofever score for TRAPS (*p* = 0.19), and the line of biologic agent (*p* = 0.36). Moreover, at univariate analysis the following clinical and laboratory manifestations during flares did not affect complete response: thoracic pain (*p* = 0.44), skin rash (*p* = 0.21), pericarditis (*p* = 0.16), pleuritis (*p* = 0.80), abdominal pain (*p* = 0.96), myalgia (*p* = 0.86), arthritis (*p* = 0.66), lymphadenitis (*p* = 0.66), ESR levels (*p* = 0.91), CRP levels (*p* = 0.94), SAA levels (*p* = 0.99).

At binary stepwise regression analysis, the following variables were significantly associated with complete response to biologic treatment: relapsing-remitting disease course (OR = 0.027, C.I. 0.001–0.841, *p* = 0.040) and the frequency of flares during the 12 months preceding biologic introduction (OR = 0.363, C.I. 0.301–0.953, *p* = 0.034); conversely, the control of clinical manifestations with corticosteroids (*p* = 0.19) and disease duration at the start of biologics (*p* = 0.36) did not result as predictive factors of complete response.

## Discussion

During the last two decades, TRAPS treatment has progressively changed along with the new insights on pathogenesis, clinical presentation, and genotype-phenotype correlations. In this perspective, the increasing awareness about genotypes more prone to develop long-term complications and the identification of subgroups of patients more likely responsive to corticosteroids and/or NSAIDs has led to a more precise definition of clinical cases requiring a more aggressive treatment approach with an early use of biologic agents (3, 4, 17, 18). In the same way, the recent advances about the pivotal role of IL-1 in TRAPS pathogenesis as well as the excellent results observed in clinical trials have increasingly enhanced the use of IL-1 antagonists at the expense of the anti-TNF-α agents (11, 19–22). On the other hand, etanercept represents the most widely employed anti-TNF-α agent for TRAPS (1, 6, 23), as the monoclonal antibodies infliximab and adalimumab have been anecdotally associated to paradoxical inflammatory attacks in such patients (8, 9, 24). Only more recently, the IL-6 tocilizumab has proven to be a further promising biologic approach capable to control disease manifestations also in cases unresponsive to TNF-α blockers; however, current literature on the role of IL-6 inhibition in TRAPS is quite limited (14).

In this scenario, the present study represents a snapshot of the biologic treatment strategies employed in TRAPS patients in the real-life context from 16 Italian tertiary centers during the last years.

Based on our results, the IL-1 inhibitors anakinra and canakinumab represent by far the most frequently employed biologics, followed by the TNF-α blocker etanercept; conversely, other anti-TNF-α agents and tocilizumab have been used only occasionally. Of note, the relatively high median age at the introduction of the first biologic (34.5 years) along with the high mean disease duration at the start of the first biologic (20.65 ± 17.57 years) reflect the remarkable diagnostic delay that affects TRAPS patients, as already highlighted especially in adult cases (25). Obviously, this represents a challenging issue in the medical history of patients with TRAPS, as a longer diagnostic delay implies a longer period of disease activity with no correct treatment and an higher probability to develop long-term organ damage, principally for patients with high-penetrance mutations and more severe phenotypes.

About three quarters of treatment courses led to complete response and about 90% of patients showed a benefit from biologic treatment, further confirming the effectiveness of current biologic agents in the treatment of TRAPS. In our study, this result was not affected by the different age at disease onset (during childhood vs. during adulthood), the penetrance of mutations and the different lines of biologics, thus suggesting that the genotype, the implications related to the different age at onset and the treatment failure to a first biologic agent do not have a significant impact on the therapeutic efficacy of biologic courses. However, 14/55 treatment courses led to only partial efficacy or even to complete failure. In this regard, among the hypotheses we could provide, the use of an inadequate biologic dosage seems to be the most pertinent. Actually, most of patients with poorer results were treated with IL-1 inhibitors; in this context, literature data show that increasing posology of IL-1 inhibitors may represent an effective and safe attempt to reach complete control in patients with different autoinflammatory conditions also when initial effectiveness does not seem promising (11, 13). Nevertheless, only a small minority of our patients underwent an increase in the dosage or in the frequency of administrations, suggesting that switching between biologics has been preferred to posology adjustments for the therapeutic management of TRAPS in real-life. On the other hand, the reverse was not true: dose de-escalation strategies were successfully attempted in almost a third of patients with complete response, pointing out that reducing the therapeutic load may be equally effective in selected patients after having reached a complete response, as also reported for other autoinflammatory conditions (13). Unfortunately, data in our possession did not allow to specify the period ranging between the start of the biologics and the reduction of the dosage.

The frequency of TRAPS exacerbations was dramatically decreased after the introduction of biologic agents if compared to the 12 months preceding the start of the treatment. This finding turns out to be true disregarding the age at disease onset, the penetrance of mutations and the biologic line of treatment. Of note, despite the highly significant decrease in the number of disease flares, whose median frequency was at most about an attack per year after biologic treatment, the start of biologic agents did not necessarily bring about a complete suppression of TRAPS exacerbations always and in any case. However, in spite of this issue, the residual flares were characterized by a significant decrease of both duration and severity. Indeed, the duration of inflammatory attacks was found significantly reduced after 12 months of biologic treatment and corresponded to a median duration of about 1 day. In addition, the AIDAI score, which currently represents the only clinimetric tool proposed to assess TRAPS activity (15), decreased in an highly significant manner after the start of treatment. Apart from an initial higher decrease of the AIDAI score observed during the first month of treatment in adult-onset TRAPS patients compared to pediatric-onset subjects, the decrease of TRAPS activity after the introduction of biologic treatment was substantially independent from patients' age at onset, penetrance of mutations and the biologic line.

Always within clinimetric assessment of patients, an increase in the ADDI, the currently used score for the evaluation of long-term TRAPS damage (16), was observed in only two patients during the whole follow-up period. On the other hand, since damage is meant as a persistent or irreversible condition, a damage index could not decrease over time. Nevertheless, if considering resolution of long-term musculoskeletal pain, in the present study the ADDI score would have decreased by one point in a not negligible percentage of patients, almost half of which carrying high-penetrance mutations.

The significant decrease in the 24 h proteinuria during the whole follow-up period in the eight treatment course with proteinuria at the start of biologics represents a further clue that corroborates the role of these agents in stopping and even improving long-term TRAPS sequelae. This is a central issue for patients who have already developed proteinuria at diagnosis and helps to clarify the real role of biotechnological therapies on the most frequent long-term complication of TRAPS, i.e., renal amyloidosis and the resulting nephrotic syndrome. Naturally, this finding is not intended to give a definitive evidence because of both the low number of patients observed and the lack of data on histological features and progression during follow-up; however, our findings reinforces the very few data currently available on the role of biologic agents on renal damage in patients with TRAPS and reflect similar data obtained from patients with other monogenic autoinflammatory diseases (26–31).

One of the goals of biological therapy is to limit the use of corticosteroids in TRAPS patients. Actually, corticosteroids alone are often beneficial in controlling clinical manifestations and aborting flares, but patients may need increasing doses or even long-time administration if frequent relapses occur. In these cases, while complete protection from the risk of reactive amyloidosis does not seem guaranteed with corticosteroids only, metasteroidal comorbidities are very frequent (32, 33). In this regard, our findings point out the remarkable steroid sparing effect of biologic treatments in TRAPS patients. Indeed, in our cohort the number of patients requiring corticosteroids was significantly reduced after the start of treatment and the daily posology significantly decreased in those cases still requiring corticosteroids during follow-up. Of note, discontinuation of corticosteroids was not found necessarily immediate, as statistical significance in the decrease of steroid use was reached at the 6-month visit from the start of biologics, but not at the 3-month assessment. Equally noteworthy, during the first months of treatment the corticosteroid sparing effect was more pronounced among patients with adult-onset TRAPS when compared to pediatric-onset cases. Conversely, according to our findings, the corticosteroid sparing effect was not influenced by the penetrance of mutations or by the different biologic lines.

The effectiveness of biologics in TRAPS is also proved by the excellent long-term cumulative DRR, as observed in Figure 8. The excellent DRR is also related to the good safety profile, with most of adverse events being not serious and not capable to lead to treatment withdrawal. In particular, *in situ* reactions were the most frequent adverse events observed and occurred in patients treated with anakinra, as often reported in literature (34). Infectious events were observed in two patients and consisted of a case of pneumonia and a case of recurrent lower urinary tract infections. As a whole, patients treated with tocilizumab have been relatively more prone to adverse events in our cohort, as pneumonia, thrombocytopenia, and galactorrhoea occurred in three cases treated with such agent. However, the very small number of patients administered tocilizumab does not allow conclusions to be drawn on this aspect. In addition, tocilizumab was always proposed after other treatment approaches and hence characterized by a more complicated clinical picture and a more recalcitrant inflammatory state.

Interestingly, despite the excellent safety profile and patients' acceptance of canakinumab, one patient developed Crohn's disease while on such treatment. To the best of our knowledge, this is the first TRAPS patient developing Crohn's disease during IL-1 inhibition. Looking into the literature, evidences about an association between TRAPS and inflammatory bowel diseases are lacking. On the other hand, inflammatory bowel diseases arisen or worsened during IL-1 inhibition have occasionally been reported (35, 36). In particular, Hügle et al. have described 3 patients developing inflammatory bowel diseases during anti-IL-1 antagonists used for systemic juvenile idiopathic arthritis (35). These authors speculated a possible interference in IL1-1α/β equilibrium in the colonic mucosa, with IL-1α acting toward inflammation and IL-1β promoting healing and enhancing repair in colonic tissue (37, 38). However, no conclusions may be drawn based on sporadic cases and whether inflammatory bowel diseases represent independent comorbidities or may be linked to IL-1 inhibition in selected cases cannot be determined at the moment.

In our cohort, a patient showed paradoxical flares after infliximab administrations, thus confirming similar experiences observed in the past (8, 9). In particular, the case reported here shows that paradoxical flares may occur even after several months of complete effectiveness of infliximab.

At binary stepwise regression analysis, the complete response to biologic treatments was associated to the relapsing-remitting disease course and to the frequency of flares/year at the start of treatment. In particular, patients suffering from a relapsing-remitting disease course were more prone to reach complete response compared to patients with a chronic disease course. In this regard, a more complex disease pattern with a persistent cytokine hypersecretion could explain why patients with a chronic course show a more difficult-to-reach complete response. However, this assumption should be determined with *ad-hoc* laboratory research studies.

Also, complete efficacy was less likely reached as the number of flares increased during the 12 months preceding biologic introduction; this finding could be related to an higher cytokine secretion in those cases presenting an higher frequency of flares per year and could maybe be solved by adjusting the posology of biologic agents employed. Nevertheless, this hypothesis should also be verified through basic research studies; in addition, the number of patients undergoing a posology escalation was very small in our cohort and statistical correction for posology adjustments was not possible.

The main limitation of this study is in the retrospective design and the low number of patients treated with specific biologic agents. For this reason, comparisons of effectiveness among different biologics used were omitted to avoid conclusions, either positive or negative, based on a relatively low number of treatment courses for any of the specific biologic agent employed. Furthermore, the limited recourse to the increase of posology in patients with partial or no response have limited the evaluation of this treatment strategy as a possible solution in selected cases. Likewise, the lack of histological data on kidney tissue makes the data on proteinuria improvement incomplete. Finally, the lack of data about the use of corticosteroids at 1-month follow-up visit and at the last assessment prevented a specific evaluation of the corticosteroid sparing effect in such time points. Beyond these limitations, the present study reflects the daily clinical practice about the use of biologic agents in referral centers along with the clinical and laboratory outcome obtained in real life after biologic introduction in TRAPS patients. Moreover, despite the small number of patients enrolled, the sample size is remarkably wide in relation to the rarity of this condition.

In conclusion, this study represents an insight into the use of biologics in patients with TRAPS as observed in real-life. Biotechnological agents have shown to be effective in controlling both clinical and laboratory manifestations, while cases not responding to a first biologic treatment found benefit from the switch to a second biologic agent. A drastic reduction in the frequency and duration of inflammatory episodes was observed, as well as a significant corticosteroid sparing effect and a remarkable containment in the progression of long-term organ damage. The effectiveness of biologic agents in TRAPS is also demonstrated by the outstanding safety profile and excellent long-term DRR. Finally, the presence of a relapsing-remitting rather than chronic disease course and the number of flares/year proved to be predictors of complete response to biologic treatments.

## Data Availability Statement

The raw data supporting the conclusions of this article will be made available by the authors, without undue reservation.

## Ethics Statement

The studies involving human participants were reviewed and approved by Ethics Committee of the University of Siena (AIDA Project; Ref. N. 14951). Written informed consent to participate in this study was provided by the participants' legal guardian/next of kin.

## Author Contributions

AV, DR, and LC conceived the study design, drafted the manuscript, and contributed to discussion. AV performed statistical analysis and drafted the manuscript. LO, MC, GL, GM, NR, AS, FL, EV, AI, LD, MJ, DM, GE, LC, SB, PP, PR, MM, OV, JS, CG, RG, LS, RM, AR, CL, and BF collected the data and contributed to discussion. All authors reviewed and approved the final manuscript.

## Conflict of Interest

LD has received consultation honoraria from SOBI, Novartis, Pfizer, Abbvie, Amgen, Biogen, Celltrion, and Roche outside of the present work. The remaining authors declare that the research was conducted in the absence of any commercial or financial relationships that could be construed as a potential conflict of interest.
